# Identification and characterisation of seed storage protein transcripts from *Lupinus angustifolius*

**DOI:** 10.1186/1471-2229-11-59

**Published:** 2011-04-04

**Authors:** Rhonda C Foley, Ling-Ling Gao, Andrew Spriggs, Lena YC Soo, Danica E Goggin, Penelope MC Smith, Craig A Atkins, Karam B Singh

**Affiliations:** 1 The WAIMR Centre for Food and Genomic Medicine, Perth, Western Australia, Australia; 2 CSIRO, Plant Industry, Private Bag 5, Wembley, Western Australia, Australia; 3 CSIRO, Plant Industry, Black Mountain, Canberra, Australia; 4 School of Biological Science, University of Sydney, Sydney, Australia; 5 School of Plant Biology, University of Western Australia, Crawley, Western Australia, Australia; 6 The UWA Institute of Agriculture, University of Western Australia, Crawley, Western Australia, Australia

**Keywords:** seed storage, seed development, legumes, allergenicity, soybean, pea, peanut, medicago

## Abstract

**Background:**

In legumes, seed storage proteins are important for the developing seedling and are an important source of protein for humans and animals. *Lupinus angustifolius *(L.), also known as narrow-leaf lupin (NLL) is a grain legume crop that is gaining recognition as a potential human health food as the grain is high in protein and dietary fibre, gluten-free and low in fat and starch.

**Results:**

Genes encoding the seed storage proteins of NLL were characterised by sequencing cDNA clones derived from developing seeds. Four families of seed storage proteins were identified and comprised three unique α, seven β, two γ and four δ conglutins. This study added eleven new expressed storage protein genes for the species. A comparison of the deduced amino acid sequences of NLL conglutins with those available for the storage proteins of *Lupinus albus *(L.), *Pisum sativum *(L.), *Medicago truncatula *(L.), *Arachis hypogaea *(L.) and *Glycine max *(L.) permitted the analysis of a phylogenetic relationships between proteins and demonstrated, in general, that the strongest conservation occurred within species. In the case of 7S globulin (β conglutins) and 2S sulphur-rich albumin (δ conglutins), the analysis suggests that gene duplication occurred after legume speciation. This contrasted with 11S globulin (α conglutin) and basic 7S (γ conglutin) sequences where some of these sequences appear to have diverged prior to speciation. The most abundant NLL conglutin family was β (56%), followed by α (24%), δ (15%) and γ (6%) and the transcript levels of these genes increased 10^3 ^to 10^6 ^fold during seed development. We used the 16 NLL conglutin sequences identified here to determine that for individuals specifically allergic to lupin, all seven members of the β conglutin family were potential allergens.

**Conclusion:**

This study has characterised 16 seed storage protein genes in NLL including 11 newly-identified members. It has helped lay the foundation for efforts to use molecular breeding approaches to improve lupins, for example by reducing allergens or increasing the expression of specific seed storage protein(s) with desirable nutritional properties.

## Background

The genus *Lupinus *from the legume family (*Fabaceae*) comprises between 200 and 600 species, of which only a few have been domesticated. *Lupinus angustifolius *(L.), also known as narrow-leaf lupin (NLL) is a grain legume crop that is gaining recognition as a potential human health food as the grain is high in protein and dietary fibre, gluten-free and low in fat and starch and thus has a very low Glycaemia Index [1]. Like other legumes, lupin crops are an asset for sustainable cropping in rotations with cereal and oil seed crops. They act as a disease break, allow more options for control of grass weeds and as nitrogen-fixing legumes, reduce the need for fertilizers, enrich the soil for subsequent crops [2]. Recently considerable interest has been directed towards legume seed proteins, with studies demonstrating nutritional, nutraceutical and health benefits [3,4]. With increased awareness in many societies of the escalating incidence of obesity and the associated risk of diabetes and cardiovascular disease, NLL is an excellent candidate as a healthy food.

The major proteins in legume seeds are storage proteins defined as any seed protein that accumulates in significant quantities, has no known function during seed development, and is rapidly hydrolysed upon germination to produce a source of N and C for the early stages of seedling growth [3,5]. Seed storage proteins have beeen classified into four families, termed 11S globulin (also known as α conglutin, legumin, legumin-like and glycinin), 7S globulin (also known as β conglutin, vicilin, convicilin and vicilin-type), 7S basic globulinalso known as γ conglutin) and 2S sulphur-rich albumin also known as δ conglutin). For simplicity, in this study we will refer to the lupin seed storage proteins as α, β, γ and δ conglutins.

Specific nutritional and pharmaceutical attributes have being assigned to lupin conglutins [3]. White lupin (*L. albus*) γ conglutin has structural similarity with xyloglucan-specific endo-beta-1,4-glucanase inhibitor proteins (XEGIPs) and Triticum aestivum xylanase inhibitor (TAXI-1) [6], and is able to bind to the hormone insulin and to the insulin-like growth factor, IGF-1 and IGF-II [7,8], and may be able to play a pharmaceutical role similar to the hypoglycaemic drug metformin [8]. NLL grain has satiety properties, because food enriched with lupin seed protein and fibre significantly influences subsequent energy intake [9]. Furthermore, bread enriched with NLL protein and fibre may help reduce blood pressure and the risk of cardiovascular disease [10,11].

As seen with the majority of edible legume grains, seed proteins from lupin species can cause allergy in a small percentage of the population [12]; 'lupin allergy' occurs either separately or together with peanut allergy or allergy to other legumes [12,13]. Peanut-lupin cross allergy has been reported in which IgE antibodies that recognise peanut allergens also cross react with NLL conglutins [14,15]. One study has proposed that all lupin conglutin families are candidate allergens [16]. However, other studies have found that α and γ conglutins are the main allergens from white lupin [17] whilst patients who were allergic specifically to NLL and not peanut had serum IgE that bound β conglutins [18].

Here we analysed NLL seed ESTs at the molecular level through the construction and sequencing of a cDNA library made from seed mRNA isolated at the major filling stage. We identified ESTs from genes belonging to each of the four conglutin families. In total 16 members were identified, eleven of which had not been described previously. These NLL conglutins are in addition to conglutins identified from the only other characterized lupin, *L. albus *for which nine conglutin sequences have been deposited in GenBank [19]. The NLL conglutin sequences were compared to each other and to other legume seed storage proteins providing an insight into the evolution of these proteins in grain legumes. We also examined the specific gene expression profiles of the NLL conglutin genes and demonstrate that the expression of each is increased significantly during seed filling. This comprehensive identification of the NLL conglutins opens up the gateway to better characterise lupin molecular biology, physiology, biochemistry and nutrition.

## Results

### Isolation of new NLL conglutin genes

**Figure 1:**
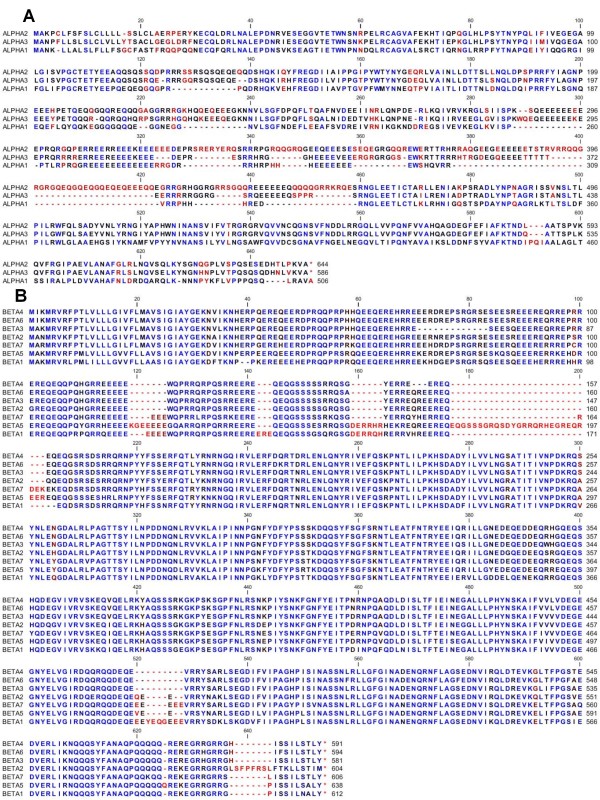
**NLL Conglutin Sequence Alignment**. Deduced amino acid alignment using CLC Genomics Workbench 3 software [20] of *L. angustifolius *(A) ALPHA [ALPHA1 Genbank:HQ670406; ALPHA2:Genbank:HQ670407; ALPHA3:Genbank:HQ670408] and (B) BETA [BETA1 Genbank:HQ670409, BETA2 Genbank:HQ670410, BETA3 Genbank:HQ670411, BETA4 Genbank:HQ670412, BETA5 Genbank:HQ670413, BETA6 Genbank:HQ670414, BETA7 Genbank:HQ670415].

**Figure 2:**
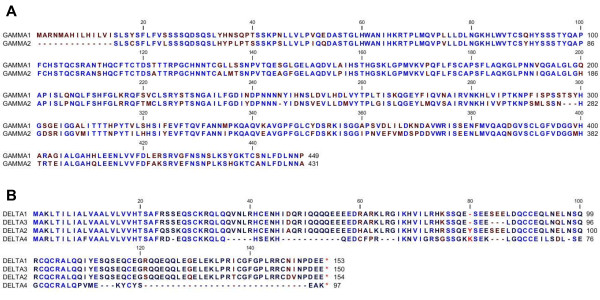
**NLL *Gamma and Delta Conglutin Sequence Alignment***. Deduced amino acid alignment using CLC Genomics Workbench 3 software [20] of *L. angustifolius *(A) GAMMA [GAMMA1 Genbank:HQ670416, GAMMA2 Genbank:HQ670417] and (B) DELTA [DELTA1 Genbank:HQ670418, DELTA2 Genbank:HQ670419, DELTA3 Genbank:HQ670420, DELTA4 Genbank:HQ670421] conglutins. Amino acids labelled blue represent those with the highest conservation among NLL congluting sequences, while those labelled red represent those with the least conservation. Dashes have been inserted to optimize alignment.

A cDNA library was constructed from NLL seed at 20-26 DAA (days after anthesis), which coincided with the major seed-filling stage. Three unique α, seven β, two γ and four δ conglutin sequences were identified after sequencing 3017 ESTs. Previously identified NLL conglutins are *ALPHA3 *[Genbank:ACN39600.1], *BETA1 *[Genbank:ACB05815.1], *BETA7 *[Genbank:ABR21771.1], *GAMMA1 *[Genbank:AAB53771.1] and *DELTA2 *[Genbank:CAA37598.1]. In addition to these five sequences there is another β conglutin [Genbank:ABR21772.1] that has identity to BETA1 between amino acids 1-445, but is truncated as it contains a premature stop codon. In this study we identified a further 11 new conglutin sequences consisting of two α, five β, one γ and three δ conglutin sequences. Within each family the sequences were aligned using the CLC Genomics Workbench 3 software [20] as shown in Figure 1 and 2. Among the α conglutin proteins, ALPHA2 [Genbank:HQ670407] and ALPHA3 [Genbank:HQ670408] were the most closely related and ALPHA1 [Genbank:HQ670406] was more divergent than ALPHA2 and ALPHA3 (Figure 1A). The seven BETA sequences [BETA1 Genbank:HQ670409, BETA2 Genbank:HQ670410, BETA3 Genbank:HQ670411, BETA4 Genbank:HQ670412, BETA5 Genbank:HQ670413, BETA6 Genbank:HQ670414, BETA7 Genbank:HQ670415] showed a high degree of identity with conservation often occurring in hydrophilic domains that were enriched in the amino acids Glu, Gln and Arg (Figure 1B). GAMMA1 [Genbank:HQ670416] and GAMMA2 [Genbank:HQ670417] share more identity with sequences from *L. albus *than between themselves (Figure 2A). DELTA1 [Genbank:HQ670418], DELTA2 [Genbank:HQ670419] and DELTA3 [Genbank:HQ670420] shared close alignment, but DELTA4, [Genbank:HQ670421] which appeared to contain a number of deletions relative to the other δ sequences did not share homology in its 3' domain (Figure 2B).

### Comparison of NLL conglutins to other legume sequences

Seed storage protein homologues from *Glycine max *(soybean), *Pisum sativum *(pea), *Arachis hypogaea *(peanut), *Medicago truncatula *and *Lupinus albus *(white lupin) that had BLAST sequence alignment scores greater than 200 when compared to any of the 16 NLL conglutin protein sequences were identified from the NCBI non-redundant protein database. These sequences were compared to each other within each family using a distance based method from the CLC Genomics Workbench 3 software [20]. Members of each family were identified from all plant species examined with the following exceptions: there were no *M. truncatula *11S globulin or 2S sulphur-rich sequences, no peanut 7S basic globulin sequences and no pea 7S basic globulin or 2S sulphur-rich sequences. The number of protein sequences identified for each species may not accurately represent the final number of members in each group. In some cases, they may under-represented as some genes may lack homology to the NLL conglutins used in this analysis, or are yet to be identified.

**Figure 3:**
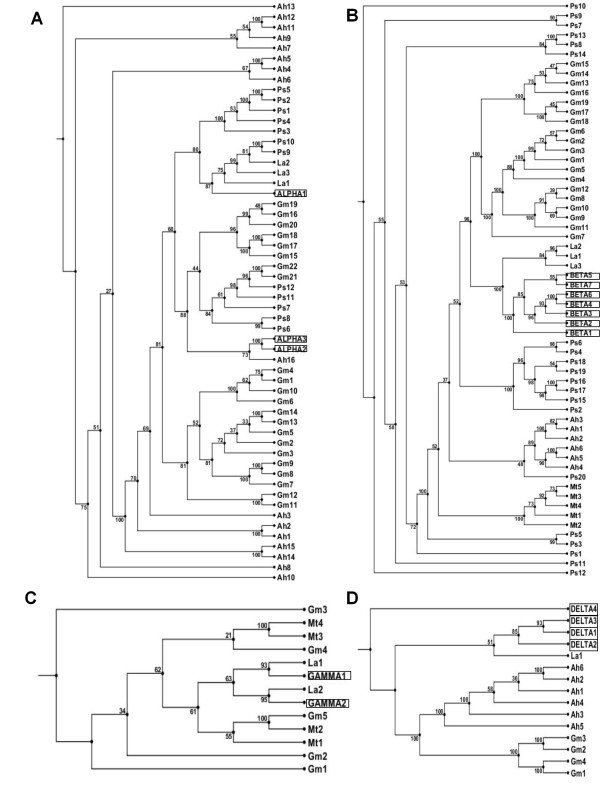
**Seed Storage protein Phylogenetic Relationships**. Phylogenetic relationships between *Arachis hypogaea *(Ah), *Glycine max *(Gm), *Medicago truncatula *(Mt), *Lupinus albus *(La), and *Pisum sativum *(Ps) conglutin-like sequences and *L. angustifolius *(A) 11S globulin (α conglutin), (B) 7S globulin (β conglutin), (C) basic 7S (γ conglutin) and (D) 2S sulphur-rich albumin (δ conglutin) deduced amino acid sequences. *L. angustifolius *conglutins are boxed for easy recognition. Identification and accession number for each protein are listed in Table 1.

Alternatively, they may be over-represented as two or more proteins may be derived from the same gene via processing [21]. Figure 3 presents the phylogenetic relationship between seed storage protein families from the six legume species studied. For simplicity all the sequences were renamed with the species initials, followed by a number. The corresponding accession numbers are listed in Table 1.

**Table 1:** *Arachis hypogaea *(Ah), *Glycine max *(Gm), *Medicago truncatula *(Mt), *Lupinus albus *(La), and *Pisum sativum *(Ps) identification and accession numbers used in Figure 3.

ALPHA homologues	BETA homologues	GAMMA homologues	DELTA homologues
▸Ah1 [gi|112380623|gb|ABI17154.1]	▸Ah1 [gi|1168390|sp|P43237.1]	▸Gm1 [gi|14549156|sp|P13917.2|	▸Ah1 [gi|31322017|gb|AAM78596.1]
▸Ah2 [gi|47933675|gb|AAT39430.1]	▸Ah2 [gi|46560478|gb|AAT00597.1]	▸Gm2 [gi|1401240|gb|AAB03390.1]	▸Ah2 [gi|46560482|gb|AAT00599.1]
▸Ah3 [gi|75253181]sp|Q647H2.1]	▸Ah3 [gi|1168391]sp|P43238.1]	▸Gm3 [gi|18543|emb|CAA34489.1]	▸Ah3 [gi|118776566|gb|ABL14268.1]
▸Ah4 [gi|37789212|gb|AAR02860.1]	▸Ah4 [gi|46560476|gb|AAT00596.1]	▸Gm4 [gi|51316037|sp|Q8RVH5.1]	▸Ah4 [gi|15418705|gb|AAK96887.1]
▸Ah5 [gi|21314465|gb|AAM46958.1]	▸Ah5 [gi|46560472|gb|AAT00594.1]	▸Gm5 [gi|255644718|gb|ACU22861.1]	▸Ah5 [gi|75114094|sp|Q647G9.1]
▸Ah6 [gi|52001221]gb|AAU21491.1]	▸Ah6 [gi|46560474|gb|AAT00595.1]	▸La1 [gi|11191819|emb|CAC16394.1]	▸Ah6 [gi|52001227|gb|AAU21494.1]
▸Ah7 [gi|199732457|gb|ACH91862.1]	▸Gm1 [gi|15425631]dbj|BAB64303.1]	▸La2 [gi|67966634|emb|CAC17729.2|	▸Gm1 [gi|5902685|sp|P19594.2]
▸Ah8 [gi|9864777|gb|AAG01363.1]	▸Gm2 [gi|68264913|dbj|BAE02726.1]	▸Mt1 [gi|87240526|gb|ABD32384.1]	▸Gm2 [gi|4097894|gb|AAD09630.1]
▸Ah9 [gi|57669861]gb|AAW56067.1]	▸Gm3 [gi|32328882|dbj|BAC78524.1]	▸Mt2 [gi|217073766|gb|ACJ85243.1]	▸Gm3 [gi|255630323|gb|ACU15518.1]
▸Ah10 [gi|118776570|gb|ABL14270.1]	▸Gm4 [gi|9967361]dbj|BAA74452.2|	▸Mt3 [gi|217069992|gb|ACJ83356.1]	▸Gm4 [gi|255627771]gb|ACU14230.1]
▸Ah11 [gi|224036293|pdb|3C3V]	▸Gm5 [gi|111278867|gb|ABH09130.1]	▸Mt4 [gi|217071718|gb|ACJ84219.1]	▸La1 [gi|80221495|emb|CAJ42100.1]
▸Ah12 [gi|3703107|gb|AAC63045.1]	▸Gm6 [gi|121286|sp|P11827.1]		
▸Ah13 [gi|5712199|gb|AAD47382.1]	▸Gm7 [gi|51247835|pdb|1UIK]		
▸Ah14 [gi|22135348|gb|AAM93157.1]	▸Gm8 [gi|74271743|dbj|BAE44299.1]		
▸Ah15 [gi|118776572|gb|ABL14271.1]	▸Gm9 [gi|14245736|dbj|BAB56161.1]		
▸Ah16 [gi|52001225|gb|AAU21493.1]	▸Gm10 [gi|121281]sp|P13916.2]		
▸Gm1 [gi|121278|sp|P11828.1]	▸Gm11 [gi|15425633|dbj|BAB64304.1]		
▸Gm2 [gi|121276|sp|P04776.2|	▸Gm12 [gi|68264915|dbj|BAE02727.1]		
▸Gm3 [gi|225651]prf||1309256A]	▸Gm13 [gi|21465631]pdb|1IPK]		
▸Gm4 [gi|27922971]dbj|BAC55937.1]	▸Gm14 [gi|21465628|pdb|1IPJ]		
▸Gm5 [gi|18615|emb|CAA26723.1]	▸Gm15 [gi|63852207|dbj|BAD98463.1]		
▸Gm6 [gi|27922973|dbj|BAC55938.1]	▸Gm16 [gi|51247829|pdb|1UIJ]		
▸Gm7 [gi|15988117|pdb|1FXZ]	▸Gm17 [gi|121282|sp|P25974.1]		
▸Gm8 [gi|42543705|pdb|1UD1]	▸Gm18 [gi|255636348|gb|ACU18513.1]		
▸Gm9 [gi|42543702|pdb|1UCX]	▸Gm19 [gi|15425635|dbj|BAB64305.1]		
▸Gm10 [gi|99909|pir||S11003]	▸La1 [gi|89994190|emb|CAI84850.2]		
▸Gm11 [gi|121277|sp|P04405.2]	▸La2 [gi|46451223|gb|AAS97865.1]		
▸Gm12 [gi|18609|emb|CAA26575.1]	▸La3 [gi|77994351]gb|ABB13526.1]		
▸Gm13 [gi|254029113|gb|ACT53400.1]	▸Mt1 [gi|87162569|gb|ABD28364.1]		
▸Gm14 [gi|254029115|gb|ACT53401.1]	▸Mt2 [gi|87162566|gb|ABD28361.1]		
▸Gm15 [gi|255224|gb|AAB23212.1]	▸Mt3 [gi|87162572|gb|ABD28367.1]		
▸Gm16 [gi|223649560|gb|ACN11532.1]	▸Mt4 [gi|87162570|gb|ABD28365.1]		
▸Gm17 [gi|4249568|dbj|BAA74953.1]	▸Mt5 [gi|87162567|gb|ABD28362.1]		
▸Gm18 [gi|121279|sp|P02858.1]	▸Ps1 [gi|290784420|emb|CBK38917.1]		
▸Gm19 [gi|10566449|dbj|BAB15802.1]	▸Ps2 [gi|117655|sp|P13915.1]		
▸Gm20 [gi|33357661]pdb|1OD5|	▸Ps3 [gi|137582|sp|P13918.2|		
▸Gm21 [gi|225440|prf||1303273A	▸Ps4 [gi|227928|prf||1713472A		
▸Gm22 [gi|169971]gb|AAA33965.1]	▸Ps5 [gi|758248|emb|CAA68708.1]		
▸La1 [gi|85361412|emb|CAI83773.2|	▸Ps6 [gi|7339551]emb|CAB82855.1]		
▸La2 [gi|62816184|emb|CAI83770.1]	▸Ps7 [gi|297170|emb|CAA47814.1]		
▸La3 [gi|62816188|emb|CAI83771.1]	▸Ps8 [gi|42414627|emb|CAF25232.1]		
▸Ps1 [gi|4218520|emb|CAA10722.1]	▸Ps9 [gi|290784430|emb|CBK38922.1]		
▸Ps2 [gi|126168|sp|P02857.1]	▸Ps10 [gi|290784424|emb|CBK38919.1]		
▸Ps3 [gi|126161]sp|P15838.1]	▸Ps11 [gi|290784426|emb|CBK38920.1]		
▸Ps4 [gi|294979728|pdb|3KSC|	▸Ps12 [gi|290784428|emb|CBK38921.1]		
▸Ps5 [gi|4379378|emb|CAA26720.1]	▸Ps13 [gi|42414629|emb|CAF25233.1]		
▸Ps6 [gi|126170|sp|P05692.1]	▸Ps14 [gi|137581]sp|P02854.1]		
▸Ps7 [gi|2578438|emb|CAA47809.1]	▸Ps15 [gi|164512526|emb|CAP06312.1]		
▸Ps8 [gi|282925|pir||S26688	▸Ps16 [gi|164512524|emb|CAP06311.1]		
▸Ps9 [gi|169124|gb|AAA33679.1]	▸Ps17 [gi|164512522|emb|CAP06310.1]		
▸Ps10 [gi|223382|prf||0801268A	▸Ps18 [gi|164512532|emb|CAP06315.1]		
▸Ps11 [gi|126171]sp|P05693.1]	▸Ps19 [gi|164512528|emb|CAP06313.1]		
▸Ps12 [gi|126169|sp|P14594.1]	▸Ps20 [gi|137579|sp|P02855.1]		

While most 11S globulin protein sequences showed the highest homology with other members in the same species, there were exceptions; for example, the NLL ALPHA1 is more homologous to sequences from white lupin (La1) and peanut than to NLL ALPHA2 or ALPHA3 (Figure 3A). In general, 7S globulin sequences showed greatest identity within species (Figure 3B). For example all NLL β conglutin-like sequences were more homologous to each other than to 7S globulin sequences from other legume species. This was also the case for soybean, *M. truncatula *and peanut. The pea seed storage protein phylogenetic relationship was more complicated, with three of the four groups being more diverged from each other than from seed storage proteins from other legumes. In the case of basic 7S sequences, the soybean basic 7S sequences were species specific with the exception of Gm5, which shared similar sequence identity with all basic 7S sequences. However this was not seen with white lupin and NLL where GAMMA1 [Genbank:HQ670416] and GAMMA2 [Genbank:HQ670417] were more similar to La1 and La2 [22] than each other (Figure 3C). Furthermore, the basic 7S Mt1 sequence was more homologous to white lupin, NLL and soybean sequences than other basic 7S sequences from *M. truncatula*. The 2S sulphur-rich albumin sequences shared the highest sequence homology within each legume species (Figure 3D), although NLL DELTA4 is quite distinct from the other NLL δ conglutin sequences.

### Changes in expression of NLL conglutins during seed development

Sequencing of ESTs from NLL seed (20-26 DAA) identified 42% as conglutins. The EST sequencing also identified expression of other major groups of genes including those encoding ribosomal proteins, protein translation factors, oleosins and seed maturation proteins.

**Figure 4:**
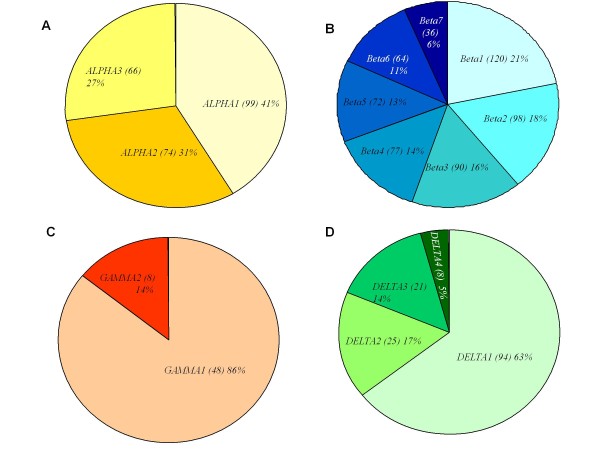
**EST Conglutin Expression**. Pie chart of relative numbers of specific members in each *L. angustifolius *conglutin family. (A) *ALPHA*, (B) *BETA*, (C) *GAMMA *and (D) *DELTA *conglutin ESTs. Total number of ESTs of each member is listed in parenthesis, followed by percentage of each total conglutin number.

The 16 unique NLL conglutin genes were used as reference sequences against all 3017 ESTs using the CLC Genomics Workbench 3 software [20]. Based on transcript levels, the most abundant conglutin family was β (56%), followed by α (24%), δ (15%) and γ (6%). The proportion (and total number) of ESTs corresponding to a particular conglutin gene within each conglutin family is presented in Figure 4, and this provides an estimate of the relative expression levels of each conglutin at 20-26 DAA.

**Figure 5:**
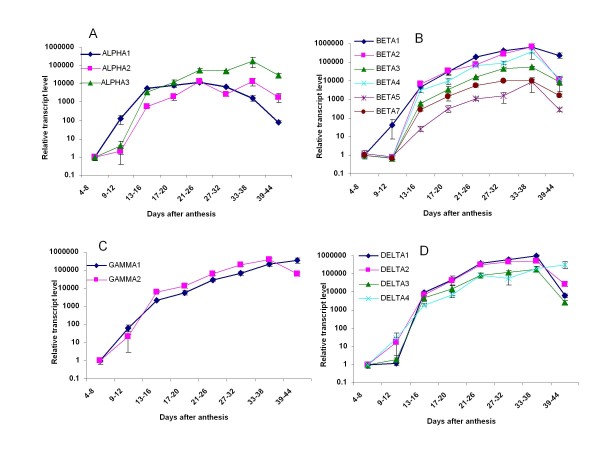
**qRT-PCR Conglutin Expression**. Relative expression of conglutin genes determined by qRT-PCR using specific primers for (A) *ALPHA*, (B) *BETA*, (C) *GAMMA *and (D) *DELTA *conglutin sequences normalised to β-tubulin. RNA was extracted from *L. angustifolius *seeds collected from different stages of development. The average and standard error of three biological replicates are plotted against the log of the relative transcript expression.

Figure 5 presents the relative expression of each conglutin gene over the time course of NLL seed development using specific primers for each conglutin gene. The time points represented seeds pooled from 4-8 DAA, 9-12 DAA, 13-16 DAA, 17-20 DAA, 21-26 DAA, 27-32 DAA, 33-38 DAA and 39-44 DAA, respectively. For each gene there was a large increase in expression ranging from 10^3 ^to 10^6 ^fold. This increase started from 4-8 DAA and in most cases the maximum was reached between 33 and 38 DAA.

### Proteomic identification of NLL conglutins and IgE binding conglutins

**Figure 6:**
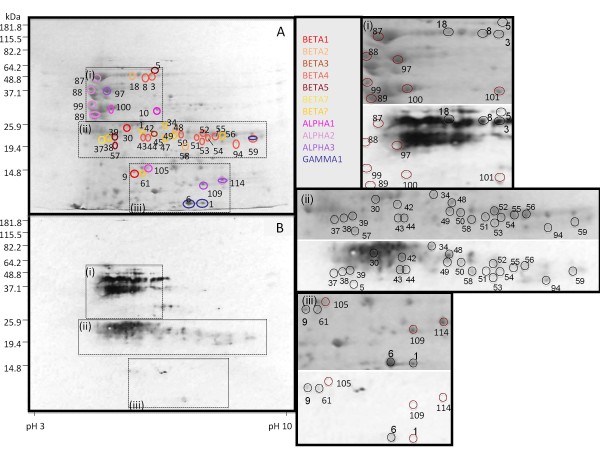
**Identification of L. angustifolius seed storage proteins**. Lupin flour proteins were separated by 2D-PAGE and (A) stained with Coomassie-blue stained or (B) blotted onto a membrane, which was probed with serum from lupin-allergic individuals, to identify potentially allergenic IgE-binding proteins. Protein spots that were either IgE-binding (spots 3 - 59, 94) or non-IgE-binding (spots 87 - 89, 97-114) were analysed by mass spectrometry, and those for which identifications were made are enclosed by ovals, with different colours corresponding to different proteins as shown in the figure. Sections of the gel and blot (boxes i, ii and iii) have been enlarged to show more detail, with Coomassie-blue stained gels on the top and IgE-binding proteins on the bottom panel for each section. In the enlarged boxes i, ii and iii spots that bind IgE are shown in black and those that do not in red. IgE binding was determined by aligning the original film and the Coomassie-blue stained gel but for 4 spots (37, 38, 51, 57) the resolution of the gel does not give a clear image of this binding. 'Beta?' indicates spots for which the form of conglutin β could not be determined.

With the availability of full-length sequences for NLL seed storage proteins derived from this work, it was possible to analyse the mass spectrometry results from the analysis of 2D blots [18] that had been probed with serum from individuals specifically allergic to lupin. Here the IgE-binding spots identified originally as β conglutin were analysed and many could be further classified into isoforms (BETA1-7). The identies of each spot are shown in Table 2 and Figure 6. BETA4 was the top match for the majority (11) of these spots. Only one spot could be unequivocally matched to BETA1, two to BETA2, one to BETA3, three to BETA5 and one to BETA7, although this does not rule out that other undetected beta isoforms may be present in these spots. In addition there maybe peptide contamination from spots that are not convincingly separated. No spots could be matched exclusively to BETA6 as for three of the spots it was not possible to distinguish between BETA6 and BETA4.

**Table 2:** Identities of lupin proteins.

Spot identity	Spot number (identity of mixed spot)
ALPHA1	89 (ALPHA2), 100, 101, 104, 105

ALPHA2	87, 88, 99, 114 (ALPHA3)

ALPHA3	97, 109

BETA1	30

BETA2	18, 48

BETA3	45

BETA4	3, 8, 43 (BETA2), 44, 50, 51, 52, 54, 55, 59 (GAMMA1), 94

BETA5	5, 39, 57

BETA6	

BETA7	37

Conglutin β Form not determined	34 (BETA3, 4, 6), 38 (BETA2, 3), 42 (BETA6, 4, 1*), 56 (BETA2, 3), 114 (ALPHA2, 3, BETA6, 4, 3*)

GAMMA1	1, 6, 59

Peptide matches for each spot are listed in Additional file 1.

* although the score for this isoform is lower than the other matches there are peptides that specifically match these forms.

There was evidence that a number of spots either contained protein from more than one β conglutin isoform or that there are other β conglutin forms that have not been identified in this study. As the seven β conglutin isoforms are conserved over the whole protein (Figure 1B), no potential epitope(s) was able to be deduced.

Three spots corresponding to GAMMA1 (spots 1, 6 and 59) were identified with the peptide coverage matching the sequence of the mature protein rather than that of the unprocessed precursor [23]. The newly-synthesised protein is first cleaved to remove a hydrophobic signal peptide and then a second time to produce large and small subunits [23,24]. Peptides identified for spot 59 matched the large subunit and spots 1 and 6 matched the small subunit which covers the C-terminus of the deduced protein (Table 2 and Additional file 1). There was no evidence of spots corresponding to the GAMMA2 protein. GAMMA1 (spot 6) showed IgE binding; however, with the higher resolution available for this analysis, it was clear that the spot was contaminated with BETA4 protein and this may explain why it appeared to bind IgE.

Mass spectrometric analysis of a number of major representative spots that did not bind IgE (spots 87-89, 99 and 100), were identified as α conglutin with ALPHA1, ALPHA2 and ALPHA3 being present (Figure 6 andTable 2).

## Discussion

This study identified 16 conglutin genes belonging to four families in NLL of which only five had been identified previously. It also significantly extended our knowledge base of seed storage proteins in lupin in general, and allowed useful comparisons with the other characterised species including *L. albus*, for which nine members have been identified in Genbank. Sequence alignment of the NLL conglutins to homologous sequences from *M. truncatula*, soybean, pea, peanut and white lupin illustrated that, in general, the strongest conservation occurred within species. In the case of β and δ conglutins, our analysis suggests that gene duplication occurred after legume speciation. This was in contrast to α and γ homologous sequences where some of these sequences were likely to have diverged prior to speciation.

The largest family in NLL was the β conglutins with seven members, while the α, γ and δ conglutin families ranged in size from two to four members. It remains to be determined if there are functional differences within each of the families. In the case of α and β conglutins, the differences between family members often involved insertions/deletions of repeated amino acid stretches of predominantly glutamic acid (E), glutamine (Q), serine (S), glycine (G) and arginine (R). These amino acids have a low hydropathy index, suggesting that the peptide regions involved are likely to be found towards the surface of the protein.

There have been a number of studies of developmental processes in legume seeds [25]. During the cell enlargement (seed-filling) phase of seed development, N accumulation and protein synthesis rely on both symbiotic N_2 _fixation and uptake of N from the soil [26]. Proteins involved in cell division are abundant during early stages of seed development, and their level decreases before the accumulation of the major storage proteins during seed-filling [27]. Our expression data provides evidence that in NLL, the conglutins began to be expressed at relatively high levels from 9-12 DAA, and peaked between 33-38 DAA, which corresponds to the seed filling stage [28]. While the general induction pattern appears similar, there are small differences between individual genes; for example *ALPHA1 *appears to both increase and then decrease earlier than the other two α genes. Whether these small variations are important in seed development remains to be determined, although it is interesting to note that phylogenetically, ALPHA2 and ALPHA3 are more closely related to each other than to ALPHA1. The similar induction pattern of all tested conglutin genes suggests that their expression may be regulated by a common mechanism and there may be a master regulator(s) to ensure overall protein quantity within the seed is maintained. Consistent with this hypothesis are the results from gene silencing of soybean β-conglycinin protein (7S globulin) which caused an increase of glycinin (11S globulin) [29]. In addition, there is likely to be fine tuning with post-transcriptional regulation of storage protein synthesis in response to N and S supply [30], and other environmental variations [31].

The deduced precursor proteins of NLL conglutins each have different molecular masses and isoelectric points (pIs) but this cannot be used to predict the mobility of the processed mature proteins on a 2D [32]. This has also been recorded for *L. albus *conglutins [33,34] where 124 polypeptide spots fell into the α, β and γ conglutin familes [35]. Analysis of the peptide coverage on the BETA spots may give some indication about the processing of β conglutin precursors to produce the mature protein. Three of the largest β conglutin spots of 48.8 kDa (spots 3, 8, 18) do not have any peptides identified that cover the N-terminal 108 amino acids suggesting that this region is cleaved in a similar manner to that for peanut Ara h1 [36]. Similarly, many of the smaller spots contain peptides only in the region from amino acid 410 to the C-terminus suggesting a second site of cleavage. These observations would need to be confirmed by N-terminal sequencing of the different spots. It is possible that differential glycosylation or some other modification may also contribute to the large number of β conglutin spots, given that many spots corresponded to the same region of a particular β conglutin form with only slight differences in size and pI (e.g. spots 52, 55 and 59).

Peanut is regarded as the most severe allergenic hazard among legume seed proteins and is the best studied with respect to allergenicity. Each of the three main peanut allergens has a homolog to lupin conglutins. Thus α conglutin corresponds to Ara h3 [37,38], β conglutin to Ara h1 [39], and δ conglutin to Ara h2 [40]. In addition, each protein has the potential to have multiple allergenic sites, for example Ara h3 contains eight distinct epitopes and most of these differ from the corresponding regions of other legume and tree-nut allergens [41]. Identification of specific allergens and their IgE binding epitopes is an important step if low-allergen traits are developed. For example, markers have been utilized to identify germplasm with reduced expression of the allergenic soybean seed P34 protein [42]. The lack of conservation of allergenic epitopes between species, and the fact that many different proteins can be allergenic makes identifying allergens across species by comparative studies difficult, and therefore the IgE-binding of each potential allergenic protein must be assessed.

Individuals allergic to peanut and lupin may react to different proteins to those that react only to lupin. A previous study based on the limited lupin conglutin sequences available at that time found that for individuals allergic to lupin but not other legumes, β conglutin was likely to be the major allergen [18]. Our results, which are based on the analysis of 16 NLL conglutin proteins, confirm and extend this earlier study and show that all β conglutin members are potential allergens, while members from other conglutin families are unlikely to be contributing to lupin specific allergenicity. At this stage it is not clear if there is a simple common epitope on the β congutins responsible for this form of allergenicity or if there are several epitopes, but it seems likely that the different forms of β conglutin share some common epitopes. When the epitope(s) that cause allergic reactions to β conglutin has been identified, breeding of varieties with reduced allergens may be possible. For example, domesticated and wild lupin could be screened for lines having reduced expression of specific allergenic conglutins or biotechnology strategies could be employed to reduce the levels of allergens in developing seeds. Already techniques using RNA interference (RNAi) to target allergen genes in peanut and tomato are showing encouraging results [43,44] and this approach can now be extended to NLL, for which transformation systems are in place [45].

## Conclusions

This study has found that *L. angustifolius *has at least 16 seed storage protein genes that fall into four families. Analysis of the expression of each gene during seed development showed that all 16 genes share similar expression patterns and are most highly expressed 33-38 days after anthesis which corresponds to the period of maximum seed filling [28]. Comparative studies to other legumes has provided insight into the evolution of these genes with evidence of gene duplication occurring after speciation in some cases. Lupin seeds, like those from other grain legumes contain allergenic proteins and our studies have identified that all seven members of the β conglutin family are potential allergens for people specifically allergic to lupins. These results provide opportunities to further characterize lupins at many levels including at the molecular biology, physiological, biochemical and nutritional levels.

## Methods

### Plant material

*L. angustifolius *(L.) *cv *Tanjil was grown in a growth cabinet at 22°C/18°C over a 14 h/12 h day/night schedule and seeds were harvested at different stages of development following anthesis (defined as DAA [days after anthesis]). Seeds from a similar developmental stage were pooled, frozen in liquid nitrogen and stored at -80°C.

### cDNA library construction and EST sequencing

A cDNA library was constructed from mRNA extracted from seeds pooled 20-26 DAA using the Invitrogen CloneMiner cDNA library construction kit based on the manufacturer's recommendations (Invitrogen, Carlsbad, CA, USA). 1.6 g of seeds yielded 15 μg mRNA. Using Invitrogen FastTract 2.0 Kit, 5 ug mRNA were used to synthesize cDNA and 160 ng of >500 bp cDNA selected on an Invitrogen column and cloned into the Invitrogen gateway vector, pDONR222. A library of ~1,000,000 clones was produced and 50 random clones were shown to have an average insert size of 1800 bp. 3017 bacterial clones from the library were sent to the Genome Center, Washington University for sequencing, with 2395 giving useful sequence information. To identify seed storage protein genes, the NCBI database was screened with the words 'conglutin', 'legumin' and 'vicilin' identifying 304 sequences. These reference sequences were then compared to the NLL EST sequences using BLAST and 1036 with homology to the reference sequences were retrieved. Using Vector NTI, the NLL conglutin ESTs were assembled. Those which were not assembled by Vector NTI, but demonstrated homology to one of the 304 sequences, were analysed individually by BLAST to determine the subgroup of best fit. Unique genes were identified within each family and the largest EST for each was sequenced in its entirety. ([ALPHA1 Genbank:HQ670406; ALPHA2:Genbank:HQ670407; ALPHA3:Genbank:HQ670408, BETA1 Genbank:HQ670409, BETA2 Genbank:HQ670410, BETA3 Genbank:HQ670411, BETA4 Genbank:HQ670412, BETA5 Genbank:HQ670413, BETA6 Genbank:HQ670414, BETA7 Genbank:HQ670415, GAMMA1 Genbank:HQ670416, GAMMA2 Genbank:HQ670417, DELTA1 Genbank:HQ670418, DELTA2 Genbank:HQ670419, DELTA3 Genbank:HQ670420 and DELTA4 Genbank:HQ670421]). The deduced amino acid sequence was determined using Vector NTI and compared by BLAST to NLL sequences in the NBCI non-redundant database [46].

### Gene expression analysis

RNA isolation and cDNA synthesis were performed using the Purescript RNA isolation kit (Qiagen, Germantown, MD, USA) according to the manufacturer's recommendation. RNA was reverse-transcribed using MLV (Promega, Madison, WI, USA) and the equivalent of 16 ng used for qRT-PCR. An EST was identified with strong homology to β-tubulin (Genbank Q39445) from *Cicer arietinum *(chickpea) (Additional file 2: Supplementary Figure S2), and was used as a control for the qRT-PCR experiments. qRT-PCR against equal amounts of total RNA demonstrated similar expression levels of β-tubulin throughout seed development confirming that it was a suitable reference gene for these studies (data not shown). Based on sequence homology unique primer sets were designed against each of the conglutin genes. Each primer set was tested against all 16 conglutin genes (in the pDONR222 vector) to confirm specificity and was also optimized for a specific annealing temperature which is listed together with the primer sequences in Additional File 3: Supplementary Figure S3. *BETA6 *was not included in the analysis as it was not possible to identify primers that could distinguish between *BETA6 *and other β conglutin sequences. qRT-PCR was carried out on the MyIQ (Biorad, Hercules CA) and analysed as previously described [47]. The results for each conglutin were normalized to the NLL β-tubulin.

### Mass spectrometric analysis of protein spots

Two dimensional gel electrophoresis of NLL *cv *Belara flour proteins and western blotting with human sera to identify potentially allergenic (IgE-binding) proteins was previously described [18]. The gels were probed with mixed serum from eight individuals allergic to lupin but not peanut. The protein spots were analysed by tandem mass spectrometry and a number of proteins were identified through peptide sequencing using Mascot MS (Matrix Science) searches. In the current study the 16 NLL unique conglutin sequences characterised were used to reanalyse the mass spectrometry data from Goggin et al. (2008) using the same search parameters in a Mascot MS/MS ion search [18].

## List of Abbreviations

NLL: narrow-leafed lupin; DAA:days after anthesis; qRT-PCR: quantitiative reverse-transcriptase polymerase chain reaction; ESTs: expressed sequence tags; RNAi: RNA interference.

## Authors' contributions

RCF was involved in the cDNA library construction, EST sequencing, bioinformatic analysis and phylogenic tree assembly. L-LG performed the qRT-PCR. AS was involved in the bioinformatic analysis, LYCS, DEG and PMCS analysed the peptide sequences for allergenicity. RCF, CAA and KBS helped design the study, and RCF, PMCS, CAA, and KBS wrote the paper. All authors discussed results and commented on the manuscript.

## Supplementary Material

Additional file 1 **Peptides identified from spots from the 2D-gel of *L. angustifolius *flour proteins**. Description: Protein spots were cut from the 2D-gel and were analysed by mass spectrometry. A Mascot MS/MS ion search of the 16 full-length conglutin proteins was used to identify spots. The peptides identified are listed in the Table with the protein matched, the percentage coverage, mascot score and the theoretical molecular mass and pI of a mature protein that included these peptides. Click here for file

Additional file 2 **Lupin beta tubulin EST sequence**. Lupin EST sequence that showed best homology to beta tubulin Q39445 from *Cicer arietinum*. Red sequences represents primer sequences designed for RT-PCR. Click here for file

Additional file 3 **Conglutin Primer sequences and annealing temperatures**. Primer sequence and annealing temperature for measuring the expression of each conglutin gene using RT-PCR. Click here for file
