# Supplementary material for: Identification and characterisation of seed storage protein transcripts from Lupinus angustifolius
Source: BMC Plant Biol. 2011 Apr 4;11:59. doi: 10.1186/1471-2229-11-59 (PMC3078879; doi:10.1186/1471-2229-11-59)
Supplement: Additional file 1 — Peptides identified from spots from the 2D-gel of L. angustifolius flour proteins. Description: Protein spots were cut from the 2D-gel and were analysed by mass spectrometry. A Mascot MS/MS ion search of the 16 full-length conglutin proteins was used to identify spots. The peptides identified are listed in the Table with the protein matched, the percentage coverage, mascot score and the theoretical molecular mass and pI of a mature protein that included these peptides. [file 1471-2229-11-59-S1.DOC]

| **Spot no.** | **Protein identification** | **Protein name** | **Sequence**  **coverage %** | **Score** | **Molecular mass**  **(M_r_^a^)** | **p*I*** | **Matched peptides** |
| --- | --- | --- | --- | --- | --- | --- | --- |
| 1 | Conglutin γ | Gamma1 | 6 | 158 | 8,612 | 4.84 | AVGPFGLCYDSR  AGIALGAHHLEENLVVFDLER |
| 3 | Conglutin β | Beta4 | 4 | 74 | 51,946 | 5.31 | FQTLYR  LENLQNYR  NPYYFSSER  EQEQQPQHGR  DQQSYFSGFSR  NFLAGSEDNVIR  FGNFYEITPNR  GLTFPGSTEDVER  LLGFGINADENQR  AIFVVLVDEGEGNYELVGIR |
| 5 | Conglutin β | Beta5 | 6 | 269 | 66,826 | 4.81 | FYDFYPSR  FGNFYEITPDR  LLGFGINADENQR  HSDADYILVVLNGR |
| 6 | Conglutin γ | Gamma1 | 11 | 312 | 8,972 | 5.83 | VGFNSNSLK  SRVGFNSNSLK  VGFNSNSLKSYGK  KISGGAPSVDLILDK  ISGGAPSVDLILDKNDAVWR  AGIALGAHHLEENLVVFDLER  AGIALGAHHLEENLVVFDLERSR |
|  | Conglutin β | Beta4 | 6 | 185 | 19,525 | 5.78 | QLDTEVK  EQVQELR  KYAQSSSR  SNKPIYSNK  VSKEQVQELR |
| 8 | Conglutin β | Beta4 | 33 | 1162 | 45,675 | 5.44 | FQTLYR  QLDTEVK  YEEIQR  EQVQELR  KYAQSSSR  QQDEQEVR  SNKPIYSNK  EQEQEQGSR  NPYYFSSER  NTLEATFNTR  EQEQGSSSSSR  QQDEQEVRR  VSKEQVQELR  DQQSYFSGFSR  NFLAGSEDNVIR  FGNFYEITPNR  GLTFPGSTEDVER  TDRLENLQNYR  QRNPYYFSSER  EEREQEQEQGSR  IVEFQSKPNTLILPK  NPYYFSSERFQTLYR  NTLEATFNTRYEEIQR  LAIPINNPGNFYDFYPSSSK  LLGFGINADENQRNFLAGSEDNVIR |
| 18 | Conglutin β | Beta2 | 13 | 528 | 40,097 | 5.07 | NPYYFSYER  FGNFYEITPDR  LLGFGINADENQR  HSDADYILVVLNGR  AIFVVVVDEGEGNYELVGIR  LAIPINNPGNFYDFYPSSTK |
| 30 | Conglutin β | Beta1 | 9 | 327 | 12,879 | 4.62 | LLGFGINANENQR  ELTFPGSIEDVER  QQDEQEEEYEQGEEEVR  NQQQSYFANAQPQQQQQR |
| 34 | Conglutin β | Beta3/4/6 | 6 | 226 | 4,655 | 6.00 | LPAGTTSYILNPDDNQNLR  LAIPINNPGNFYDFYPSSSK |
| 37 | Conglutin β | Beta7 | 7 | 291 | 15,308 | 4.23 | FGNFYEITPER  NFLAGSEDNVISQLDR  AIFIVVVDEGEGNYELVGIR |
| 38 | Conglutin β | Beta2/3 | 5 | 192 | 8,657 | 4.39 | LLGFGINADENQR  AIFVVVVDEGEGNYELVGIR |
| 39 | Conglutin β | Beta5 | 11 | 436 | 19,075 | 4.48 | GKPSESGPFNLR  NFLAGSEDNVIR  FGNFYEITPDR  LLGFGINADENQR  ELIFPGSAEDVER  AIFVIVVDEGEGNYELVGIR |
| 42 | Conglutin β | Beta6/4 | 10 | 337 | 17,284 | 6.18 | FGNFYEITPNR  LPAGTTSYILNPDDNQNLR  LAIPINNPGNFYDFYPSSSK  *AIFVVLVDEGEGNYELVGIR* |
|  | Conglutin β | Beta1 | 13 | 332 | 33,421 | 4.77 | LLGFGINANENQR  ELTFPGSIEDVER  **LPAGTTSYILNPDDNQNLR**  AIFIVVVDEGEGNYELVGIR |
| 43 | Conglutin β | Beta4 | 10 | 362 | 18,909 | 4.60 | NFLAGSEDNVIR  FGNFYEITPNR  GLTFPGSTEDVER  LLGFGINADENQR  NQQQSYFANAQPQQQQQR |
|  | Conglutin β | Beta2/3 | 9 | 361 | 14,801 | 4.57 | **NFLAGSEDNVIR**  **LLGFGINADENQR**  AIFVVVVDEGEGNYELVGIR  **NQQQSYFANAQPQQQQQR** |
| 44 | Conglutin β | Beta4 | 15 | 468 | 16,380 | 4.44 | NFLAGSEDNVIR  FGNFYEITPNR  GLTFPGSTEDVER  LLGFGINADENQR  NPQAQDLDISLTFIEINEGALLLPHYNSK |
| 45 | Conglutin β | Beta3 | 15 | 354 | 22,692 | 4.67 | FGNFYEITPDR  LPAGTTSYILNPDDNQNLR  AIFVVVVDEGEGNYELVGIR  LAIPINNPGNFYDFYPSSSK  NPQAQDLDISLTFIEINEGALLLPHYNSK |
| 48 | Conglutin β | Beta2 | 17 | 723 | 33,618 | 5.29 | LENLQNYR  NTLEATFNTR  NPYYFSYER  DQQSYFNGFSR  HSDADYILVVLNGR  LPAGTTSYILNPDDNQNLR  AIFVVVVDEGEGNYELVGIR  LAIPINNPGNFYDFYPSSTK |
| 50 | Conglutin β | Beta4 | 11 | 322 | 20,600 | 5.17 | EQVQELR  NFLAGSEDNVIR  FGNFYEITPNR  GLTFPGSTEDVER  LLGFGINADENQR |
| 51 | Conglutin β | Beta4 | 11 | 371 | 23,129 | 5.54 | EQVQELR  NFLAGSEDNVIR  FGNFYEITPNR  GLTFPGSTEDVER  LLGFGINADENQR  NQQQSYFANAQPQQQQQR |
| 52 | Conglutin β | Beta4 | 8 | 311 | 18,909 | 4.60 | FGNFYEITPNR  GLTFPGSTEDVER  LLGFGINADENQR  NQQQSYFANAQPQQQQQR |
| 54 | Conglutin β | Beta4 | 13 | 360 | 20,600 | 5.17 | EQVQELR  QQDEQEVR  NFLAGSEDNVIR  FGNFYEITPNR  GLTFPGSTEDVER  LLGFGINADENQR |
| 55 | Conglutin β | Beta4 | 13 | 441 | 16,380 | 4.44 | FGNFYEITPNR  GLTFPGSTEDVER  LLGFGINADENQR  NPQAQDLDISLTFIEINEGALLLPHYNSK |
| 56 | Conglutin β | Beta2/3 | 6 | 309 | 9,973 | 4.38 | NFLAGSEDNVIR  LLGFGINADENQR  AIFVVVVDEGEGNYELVGIR |
| 57 | Conglutin β | Beta5 | 16 | 485 | 19,456 | 4.48 | NFLAGSEDNVIR  FGNFYEITPDR  LLGFGINADENQR  ELIFPGSAEDVER  AIFVIVVDEGEGNYELVGIR  NQQQSYFANAQPQQQQQQR  LSEGDILVIPAGHPLSINASSNLR |
| 59 | Conglutin β | Beta4 | 10 | 318 | 16,380 | 4.44 | NFLAGSEDNVIR  FGNFYEITPNR  GLTFPGSTEDVER  LLGFGINADENQR |
|  | Conglutin γ | Gamma1 | 8 | 257 | 23,038 | 7.74 | QGEYFIQVNAIR  TPLMQVPLLLDLNGK  VPQFLFSCAPSFLAQK |
| 87 | Conglutin α | Alpha2 | 34 | 1492 | 48,756 | 4.85 | GLSIISPK  EQQQGAGGR  RGLSIISPK  LNALEPDNR  EQQQGAGGRR  GKHQQEQEEEGK  EEEEEEQQQQQGR  SQEEEEEEEEEPR  SQSQEQEQQDSHQK  LQLAQLSKCLGASLQR  AQGEEGEEEEEETSTR  QGEEQEEESESEQEGR  SQEEEEEEEEEPRQR  RAQGEEGEEEEEETSTR  VESEGGVTETWNSNRPELR  SGQQREEEEEEQQQQQGR  LVAINLLDTTSLLNQLDPSPR  QGEEQEEESESEQEGRGQQR  SQSQEQEQQDSHQKIQYFR  EGDIIAIPPGIPYWTYNYGEQR  FYIAGNPEEEHPETQEQQGQQR  RFYIAGNPEEEHPETQEQQGQQR  HQQEQEEEGKNNVLSGFDPQFLTQAFNVDEEIINR |
| 88 | Conglutin α | Alpha2 | 15 | 516 | 24,842 | 4.79 | VESEGGVTETWNSNRPELR  LVAINLLDTTSLLNQLDPSPR  EGDIIAIPPGIPYWTYNYGEQR  FYIAGNPEEEHPETQEQQGQQR  NNVLSGFDPQFLTQAFNVDEEIINR |
| 89 | Conglutin α | Alpha1 | 27 | 722 | 28,249 | 4.53 | EGSIVEVK  VISPPTLRPR  LNALEPDNSVK  NDDREGSIVEVK  QGREEEEEEEEEEEER  RPFYTNAPQEIYIQQGR  RFYLSGNQEQEFLQYQQK  LNALEPDNSVKSEAGTIETWNPNNDQLR  EGGQGQQQEGGNEGGNVLSGFNDEFLEEAFSVDR  EGGQGQQQEGGNEGGNVLSGFNDEFLEEAFSVDREIVR |
|  | Conglutin α | Alpha2 | 24 | 636 | 29,401 | 4.86 | GLSIISPK  LQNPDER  EQQQGAGGR  RGLSIISPK  SQEEEEEEEEEPR  SQSQEQEQQDSHQK  SQEEEEEEEEEPRQR  RAQGEEGEEEEEETSTR  LVAINLLDTTSLLNQLDPSPR  EGDIIAIPPGIPYWTYNYGEQR  RFYIAGNPEEEHPETQEQQGQQR  NNVLSGFDPQFLTQAFNVDEEIINR  HQQEQEEEGKNNVLSGFDPQFLTQAFNVDEEIINR |
| 94 | Conglutin β | Beta4 | 7 | 275 | 12,860 | 4.56 | FGNFYEITPNR  LLGFGINADENQR  LSEGDIFVIPAGHPISINASSNLR |
| 97 | Conglutin α | Alpha3 | 8 | 299 | 25,613 | 5.22 | LNALEPDNR  FYLAGNPEEEYPETQQQR  NNILSGFDPQFLSQALNIDEDTVHK |
| 99 | Conglutin α | Alpha2 | 12 | 455 | 13,077 | 4.59 | LVAINLLDTTSLLNQLDPSPR  EGDIIAIPPGIPYWTYNYGEQR  FYIAGNPEEEHPETQEQQGQQR  NNVLSGFDPQFLTQAFNVDEEIINR |
| 100 | Conglutin α | Alpha1 | 18 | 489 | 25,021 | 4.84 | VISPPTLRPR  GIFGLIFPGCR  SEAGTIETWNPNNDQLR  RPFYTNAPQEIYIQQGR  FYLSGNQEQEFLQYQQK  EGGQGQQQEGGNEGGNVLSGFNDEFLEEAFSVDR |
| 101 | Conglutin α | Alpha1 | 11 | 126 | 17,369 | 4.42 | RPFYTNAPQEIYIQQGR  FYLSGNQEQEFLQYQQK  EGGQGQQQEGGNEGGNVLSGFNDEFLEEAFSVDR |
| 104 | Conglutin α | Alpha1 | 4 | 122 | 13,769 | 4.78 | TLTSLDFPILR  ALPLDVVAHAFNLDR |
| 105 | Conglutin α | Alpha1 | 7 | 229 | 13,768 | 4.78 | TLTSLDFPILR  ALPLDVVAHAFNLDR  TNDIPQIAALAGLTSSIR |
| 109 | Conglutin α | Alpha3 | 14 | 436 | 15,764 | 9.16 | ADLYNPTAGR  LSLNQVSELK  GIPAEVLANAFR  VQVVNSQGNSVFNDDLR  NGIYAPHWNINANSVIYVIR  GQLLVVPQNFVVAHQAGDEGFEFIAFK |
| 114 | Conglutin α | Alpha2 | 15 | 764 | 16,896 | 9.80 | LNQVSQLK  ADLYNPNAGR  TNDLAATSPVK  LLENIAKPSR  ISSVNSLTLPILR  GIPAEVLANAFGLR  WFQLSADYVNLYR  LLENIAKPSRADLYNPNAGR  GIPAEVLANAFGLRLNQVSQLK  GQLLVVPQNFVVAHQAGDEGFEFIAFK  RGQLLVVPQNFVVAHQAGDEGFEFIAFK  GQLLVVPQNFVVAHQAGDEGFEFIAFKTNDLAATSPVK |
|  | Conglutin α | Alpha3 | 9 | 334 | 7,157 | 6.78 | LSLNQVSELK  TNDQATTSPLK  GIPAEVLANAFR  GQLLVVPQNFVVAHQAGDEGFEFIAFK  RGQLLVVPQNFVVAHQAGDEGFEFIAFK |
|  | Conglutin β | Beta6/4 | 9 | 321 | 34,586 | 5.12 | YEEIQR  QQDEQEVR  NTLEATFNTR  LLGFGINADENQR  IVEFQSKPNTLILPK  NTLEATFNTRYEEIQR |

**Additional File 4.** Peptides identified for spots from 2D-gel of *L. angustifolius* flour proteins.

^a^Theoretical data based on the peptides matched using the Mascot algorithm

Bold text: Peptide seen in both top and second match

Italic text: The score for these peptides is not significant
