# Supplementary material for: Identification and characterisation of seed storage protein transcripts from Lupinus angustifolius
Source: BMC Plant Biol. 2011 Apr 4;11:59. doi: 10.1186/1471-2229-11-59 (PMC3078879; doi:10.1186/1471-2229-11-59)
Supplement: Additional file 2 — Lupin beta tubulin EST sequence. Lupin EST sequence that showed best homology to beta tubulin Q39445 from Cicer arietinum. Red sequences represents primer sequences designed for RT-PCR. [file 1471-2229-11-59-S2.PPT]

## Slide 1
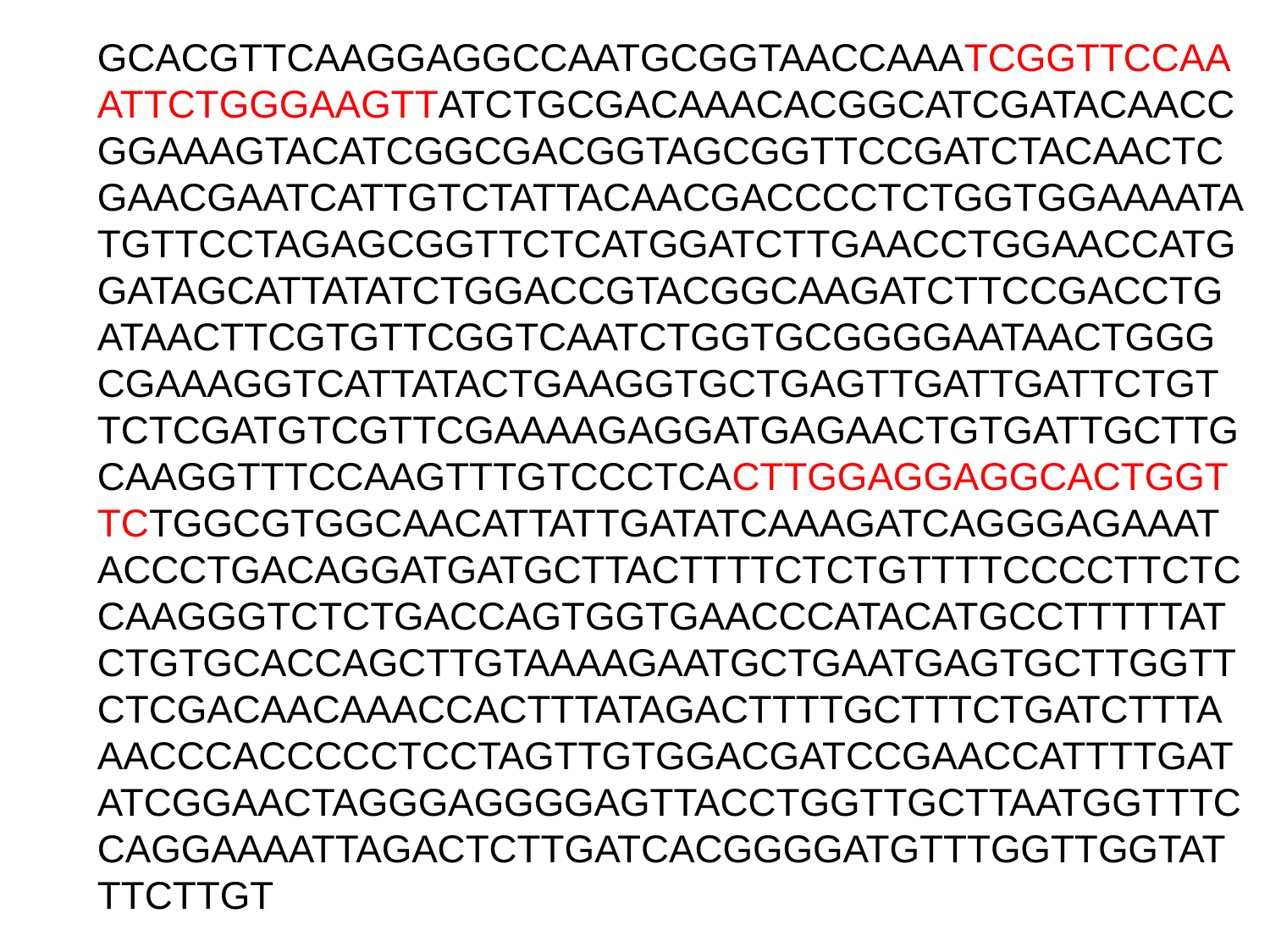

GCACGTTCAAGGAGGCCAATGCGGTAACCAAATCGGTTCCAAATTCTGGGAAGTTATCTGCGACAAACACGGCATCGATACAACCGGAAAGTACATCGGCGACGGTAGCGGTTCCGATCTACAACTCGAACGAATCATTGTCTATTACAACGACCCCTCTGGTGGAAAATATGTTCCTAGAGCGGTTCTCATGGATCTTGAACCTGGAACCATGGATAGCATTATATCTGGACCGTACGGCAAGATCTTCCGACCTGATAACTTCGTGTTCGGTCAATCTGGTGCGGGGAATAACTGGGCGAAAGGTCATTATACTGAAGGTGCTGAGTTGATTGATTCTGTTCTCGATGTCGTTCGAAAAGAGGATGAGAACTGTGATTGCTTGCAAGGTTTCCAAGTTTGTCCCTCACTTGGAGGAGGCACTGGTTCTGGCGTGGCAACATTATTGATATCAAAGATCAGGGAGAAATACCCTGACAGGATGATGCTTACTTTTCTCTGTTTTCCCCTTCTCCAAGGGTCTCTGACCAGTGGTGAACCCATACATGCCTTTTTATCTGTGCACCAGCTTGTAAAAGAATGCTGAATGAGTGCTTGGTTCTCGACAACAAACCACTTTATAGACTTTTGCTTTCTGATCTTTAAACCCACCCCCTCCTAGTTGTGGACGATCCGAACCATTTTGATATCGGAACTAGGGAGGGGAGTTACCTGGTTGCTTAATGGTTTCCAGGAAAATTAGACTCTTGATCACGGGGATGTTTGGTTGGTATTTCTTGT
