# Supplementary material for: Identification and characterisation of seed storage protein transcripts from Lupinus angustifolius
Source: BMC Plant Biol. 2011 Apr 4;11:59. doi: 10.1186/1471-2229-11-59 (PMC3078879; doi:10.1186/1471-2229-11-59)
Supplement: Additional file 3 — Conglutin Primer sequences and annealing temperatures. Primer sequence and annealing temperature for measuring the expression of each conglutin gene using RT-PCR. [file 1471-2229-11-59-S3.PPT]

## Slide 1
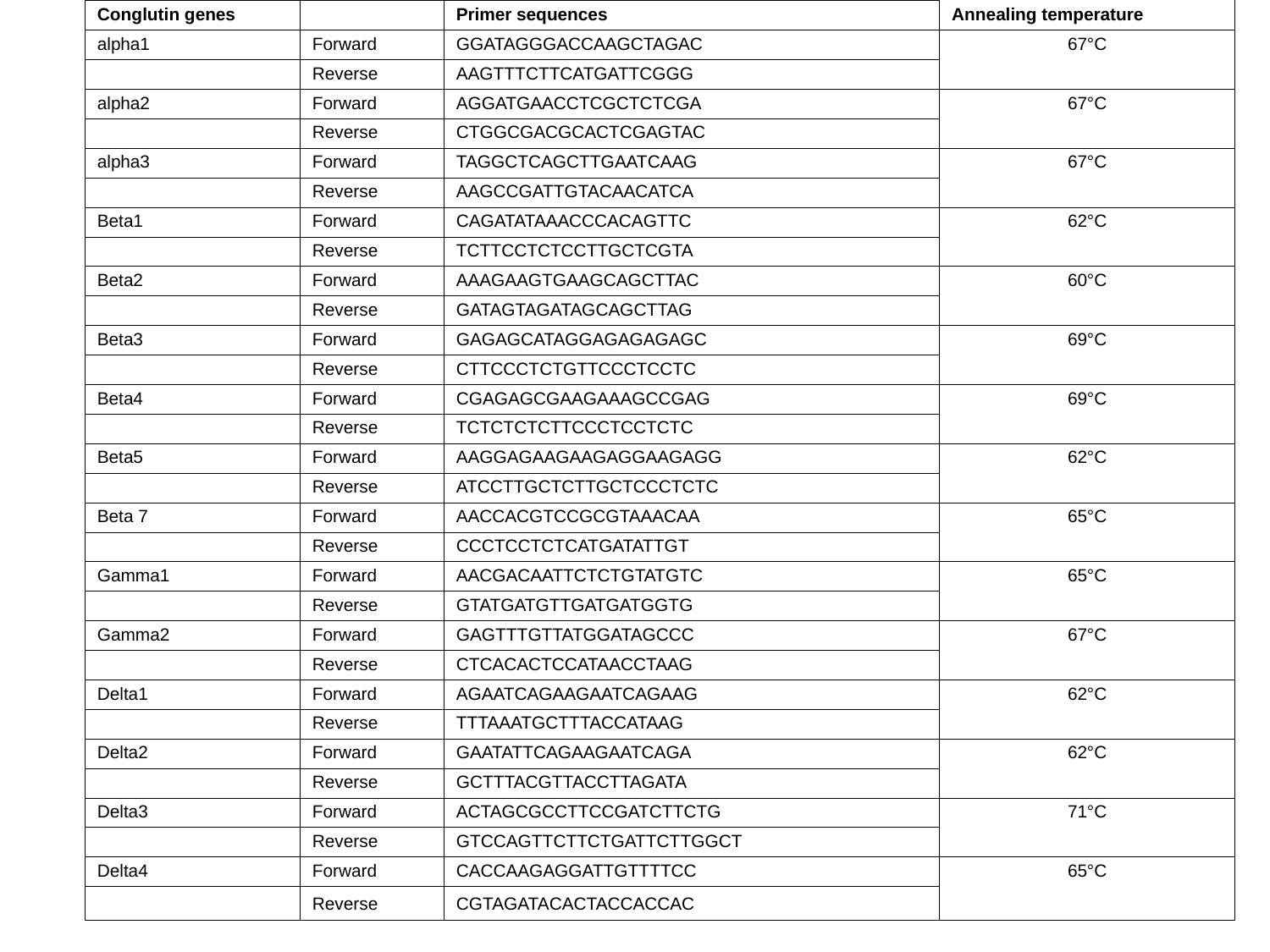

| Conglutin genes | | Primer sequences | Annealing temperature |
| --- | --- | --- | --- |
| alpha1 | Forward | GGATAGGGACCAAGCTAGAC | 67°C |
| | Reverse | AAGTTTCTTCATGATTCGGG | |
| alpha2 | Forward | AGGATGAACCTCGCTCTCGA | 67°C |
| | Reverse | CTGGCGACGCACTCGAGTAC | |
| alpha3 | Forward | TAGGCTCAGCTTGAATCAAG | 67°C |
| | Reverse | AAGCCGATTGTACAACATCA | |
| Beta1 | Forward | CAGATATAAACCCACAGTTC | 62°C |
| | Reverse | TCTTCCTCTCCTTGCTCGTA | |
| Beta2 | Forward | AAAGAAGTGAAGCAGCTTAC | 60°C |
| | Reverse | GATAGTAGATAGCAGCTTAG | |
| Beta3 | Forward | GAGAGCATAGGAGAGAGAGC | 69°C |
| | Reverse | CTTCCCTCTGTTCCCTCCTC | |
| Beta4 | Forward | CGAGAGCGAAGAAAGCCGAG | 69°C |
| | Reverse | TCTCTCTCTTCCCTCCTCTC | |
| Beta5 | Forward | AAGGAGAAGAAGAGGAAGAGG | 62°C |
| | Reverse | ATCCTTGCTCTTGCTCCCTCTC | |
| Beta 7 | Forward | AACCACGTCCGCGTAAACAA | 65°C |
| | Reverse | CCCTCCTCTCATGATATTGT | |
| Gamma1 | Forward | AACGACAATTCTCTGTATGTC | 65°C |
| | Reverse | GTATGATGTTGATGATGGTG | |
| Gamma2 | Forward | GAGTTTGTTATGGATAGCCC | 67°C |
| | Reverse | CTCACACTCCATAACCTAAG | |
| Delta1 | Forward | AGAATCAGAAGAATCAGAAG | 62°C |
| | Reverse | TTTAAATGCTTTACCATAAG | |
| Delta2 | Forward | GAATATTCAGAAGAATCAGA | 62°C |
| | Reverse | GCTTTACGTTACCTTAGATA | |
| Delta3 | Forward | ACTAGCGCCTTCCGATCTTCTG | 71°C |
| | Reverse | GTCCAGTTCTTCTGATTCTTGGCT | |
| Delta4 | Forward | CACCAAGAGGATTGTTTTCC | 65°C |
| | Reverse | CGTAGATACACTACCACCAC | |
